# P02.91. The effect of a standardized massage application on spinal stiffness in asymptomatic subjects

**DOI:** 10.1186/1472-6882-12-S1-P147

**Published:** 2012-06-12

**Authors:** GN Kawchuk, N Prasad, R Chamberlain, A Klymkiv, L Peter

**Affiliations:** 1 University of Alberta, Edmonton, Canada

## Purpose

It has been speculated that massage may exert a therapeutic effect by reducing tissue stiffness through viscoelastic mechanisms. To date, no studies have evaluated spinal stiffness post-massage. The objective of this study was to quantify changes in spinal stiffness following a standardized application of massage in asymptomatic subjects.

## Methods

Asymptomatic subjects were randomized to an experimental group (n=20) that received lumbar spine massage or a control group (n=18) that did not receive massage but read quietly. To standardize massage application in experimental subjects, a mechanical device was used with application pressure determined by the subject’s comfort level. Spinal stiffness was measured at the third lumbar vertebra using a validated indentation device. Pre- and post- massage stiffness values were compared using a Generalized Linear Model (alpha = 0.05).

## Results

Our analysis revealed no statistically significant difference in lumbar stiffness measures between subjects who received massage and those who did not (p > 0.05).

## Conclusion

In asymptomatic subjects, spinal stiffness did not change significantly in subjects who received a standardized mechanical massage compared to subjects where massage was withheld. This observation is consistent with other studies that have reported an absence of change in spinal stiffness following interventions applied in asymptomatic subjects (e.g. manipulation). Notably, spinal stiffness has been shown to decrease in symptomatic subjects following spinal manipulation. Given the results of other studies using mechanical devices to apply forces to musculoskeletal tissues, we do not expect that having massage applied by a therapist would have altered the conclusion of this study. Moreover, we speculate that in asymptomatic subjects, stiffness values are near a minimal value which creates a floor effect.

This work was supported in part by the Canada Research Chairs program (support for GN Kawchuk) and the Natural Sciences and Engineering Research Council of Canada (operating costs).

